# Characterization of *in Vitro* Modified Human Very Low-Density Lipoprotein Particles and Phospholipids by Capillary Electrophoresis

**DOI:** 10.3390/ijms131216400

**Published:** 2012-12-03

**Authors:** Yi-Ning Liu, Ting-Yu Shu, Huai-Guang Xie, Wei-Ting Lai, Yi-Han Liao, Mei-Yu Su, You-Sian Lin, Yen-Yi Chen, Yi-Jyun Lin, Chin-Pong Chong, Mine-Yine Liu

**Affiliations:** Department of Chemistry, National Changhua University of Education, Changhua 50058, Taiwan; E-Mails: ynliu@cc.ncue.edu.tw (Y.-N.L.); tyshu@cc.ncue.edu.tw (T.-Y.S.); hgxie@cc.ncue.edu.tw (H.-G.X.); wtlai@cc.ncue.edu.tw (W.-T.L.); yhliao@cc.ncue.edu.tw (Y.-H.L.); mysu@cc.ncue.edu.tw (M.-Y.S.); yslin@cc.ncue.edu.tw (Y.-S.L.); yychen@cc.ncue.edu.tw (Y.-Y.C.); yjlin@cc.ncue.edu.tw (Y.-J.L.); cpchong@cc.ncue.edu.tw (C.-P.C.)

**Keywords:** very low-density lipoprotein, phospholipids, solid phase extraction, capillary zone electrophoresis, micellar electrokinetic chromatography, *in vitro* oxidation, *in vitro* glycation

## Abstract

A simple capillary zone electrophoresis (CZE) method was used to characterize human very low-density lipoprotein (VLDL) particles for four healthy donors. One major peak was observed for native, *in vitro* oxidized and glycated VLDL particles. The effective mobilities and peak areas of the capillary electrophoresis (CE) profiles showed good reproducibility and precision. The mobility of the oxidized VLDL peak was higher than that of the native VLDL. The mobility of the glycated VLDL peak was similar to that of the native VLDL. Phospholipids isolated from VLDL particles were analyzed by our recently developed micellar electrokinetic chromatography (MEKC) with a high-salt stacking method. At absorbance 200 nm, the native VLDL phospholipids showed a major peak and a minor peak for each donor. For oxidized VLDL phospholipids, the area of the major peak reduced for three donors, possibly due to phospholipid decomposition. For glycated VLDL phospholipids, the peak mobilities were more positive than native VLDL phospholipids for two donors, possibly due to phospholipid-linked advanced glycation end products (AGEs). Very interestingly, at absorbance 234 nm, the major peak of oxidized VLDL phospholipids was resolved as two peaks for each donor, possibly due to conjugated dienes formed upon oxidation.

## 1. Introduction

Hepatocytes synthesize very low-density lipoprotein (VLDL) particles. VLDL particles are large and heterogeneous (diameter: 30–80 nm; density: 0.95–1.006 g/mL). Each hepatocyte synthesized VLDL particle contains an apo B-100 protein and is enriched in triglyceride. VLDL particles are released into the plasma and are partially hydrolyzed by lipoprotein lipase (LPL) in the periphery. Most of the hydrolyzed VLDL particles are re-uptaken by hepatocytes through the apo B/E receptor, and about 10%–20% of the hydrolyzed VLDL particles are further metabolized into low-density lipoprotein (LDL) particles [1].

Metabolic syndrome (MS) and Type 2 diabetes (T2D) have reached epidemic proportions in the countries with a Western life-style [2]. The characteristics of MS and T2D is dyslipidemia, which shows high levels of VLDL triglyceride, low levels of high-density lipoprotein (HDL) and increased numbers of small, dense LDL particles [3–5]. The enhanced circulating levels of VLDL triglyceride result from overproduction and decreased re-uptake of VLDL particles by liver [6]. However, under the hyperglycemic and oxidative stress conditions of MS and T2D, how the VLDL particles are modified is not well defined. How the modified VLDL particles influence their further hydrolysis into LDL particles is not well understood either.

Previously, capillary isotachophoresis has been used to analyze VLDL particles [7–16]. A simple capillary electrophoresis (CE) method, which included detergent in the CE separation buffer has also been used to analyze VLDL particles [17]. Microchip electrophoresis has been used to analyze human lipoprotein fractions including VLDL, LDL and HDL [18,19]. Previously, liquid chromatography/electrospray-ionisation/mass spectrometry (LC/ESI/MS) analysis has been used to identify VLDL phospholipids from humans and rats [20,21]. Matrix-assisted laser desorption/ionization-Time Of Flight (MALDI-TOFI analysis has also been used to analyze human VLDL phospholipids [22]. CE has many advantages for analyzing biomolecules over other analytical methods including high speed, high sensitivity, and minute volumes of buffer and sample needed. So far, the investigation of VLDL particles and phospholipids by CE is less explored.

The aim of this study was to characterize human VLDL particles and phospholipids under *in vitro* hyperglycemic and oxidative stress conditions by CE. Native VLDL particles isolated from healthy donors were oxidized *in vitro* by Cu^2+^ (2.5 μM) or glycated *in vitro* by glucose (60 mM). The native and modified VLDL particles were analyzed by a simple capillary zone electrophoresis (CZE) method, which had previously been developed by us. The VLDL phospholipids were analyzed by a micellar electrokinetic chromatograph (MEKC) with high-salt stacking method, which had also previously been developed by us. To the best of our knowledge, this study demonstrates for the first time the analysis of *in vitro* oxidized and glycated human VLDL by CZE and MEKC methods. The combination of CZE and MEKC methods might have the potential to analyze *in vivo* human VLDL particles and phospholipids associated with MS and T2D in the future. Furthermore, this study might also provide insight into the relationship of biochemically modified VLDL particles and their pro-atherogenic properties.

## 2. Results and Discussion

### 2.1. CZE Profiles of Native, *in vitro* Oxidized and Glycated VLDL Particles

**Figure 1:**
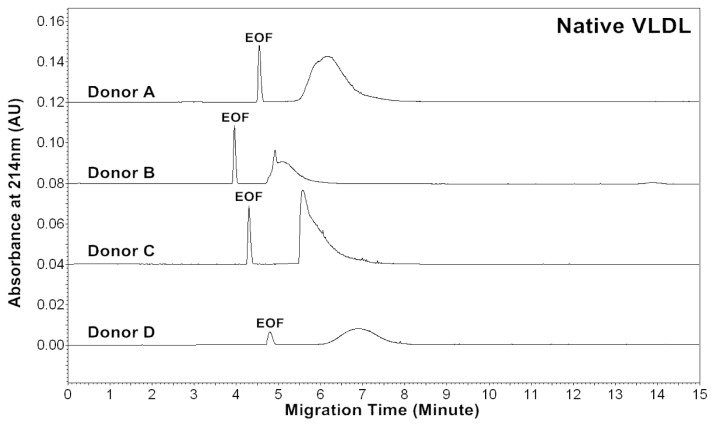
Electropherograms of VLDL particles of four healthy donors. The buffer of VLDL isolated by ultracentrifugation was exchanged from KBr solution to 5 mM sodium phosphate, pH 7.40 using a 100 kDa ultrafiltration filter (MW cut-off: 100,000). For VLDL sample introduction, a pressure of 0.5 psi and a 4 s injection were used. The capillary electrophoresis (CE) separation buffer was 5 mM sodium phosphate, pH 7.40. A voltage of 16 kV was applied and the capillary was kept at 25 °C.

**Figure 2:**
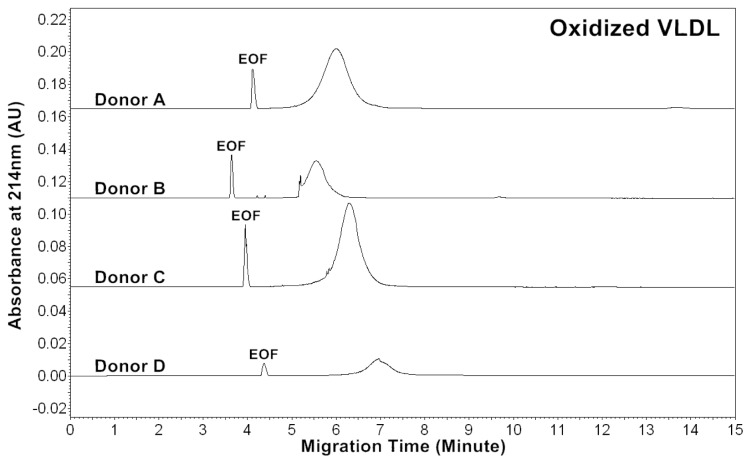
Electropherograms of *in vitro* oxidized VLDL particles of four healthy donors. VLDL was oxidized by 2.5 μM Cu^2+^ in phosphate buffered saline (PBS) buffer at 37 °C for 18 h, and the buffer was then exchanged to 5 mM sodium phosphate, pH 7.40 using a 100 kDa ultrafiltration filter (MW cut-off: 100,000). CE conditions are the same as in Figure 1.

**Figure 3:**
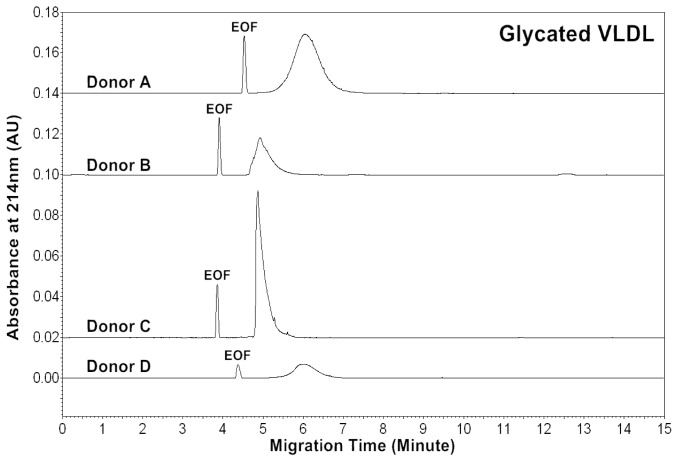
Electropherograms of *in vitro* glycated VLDL particles of four healthy donors. VLDL was incubated with 60 mM glucose in PBS buffer at 37 °C for 5 days, and the buffer was then exchanged to 5 mM sodium phosphate, pH 7.40 using a 100 kDa ultrafiltration filter (MW cut-off: 100,000). CE conditions are the same as in Figure 1.

The CZE profiles of VLDL particles for four healthy subjects are shown in Figures 1–3. For each donor, native, *in vitro* oxidized or glycated VLDL particles showed a major peak.

Tables 1–3 show the average effective mobilities (μ_eff_), peak areas (A_214_), average peak area ratios (A_234_/A_214_ and A_280_/A_214_) and CV(%) for native, oxidized and glycated VLDL particles, respectively. Eletrophoretic mobility of a particle can be expressed as:

(1) $$\mu_{\text{ep}}=(1/t)\times (L/V)$$

where l is the migration distance of the particle from the inlet to the detector, *t* is the migration time, *L* is the total length of the capillary, and *V* is the applied voltage. Electroosmotic flow (EOF) plays a key role in CZE. The effective mobility of a particle is determined by the sum of its own electrophoretic mobilities and of that of the EOF:

(2) $$\mu_{\text{eff}}=\mu_{\text{ep}}+\mu_{\text{eof}}$$

For each donor, the experiments were repeated four times to obtain the measurements. Measurements by absorbances at 214, 234 and 280 nm allowed us to estimate lipids, conjugated dienes and protein contents in VLDL particles, respectively. The data suggested that the VLDL electropherograms were highly reproducible with good precisions of effective mobilities and peak areas.

Patients of cardiovascular disease are often under oxidative stress. To monitor *in vivo* VLDL particles under oxidative stress, native VLDL particles were oxidized *in vitro* by Cu^2+^ (2.5 μM), and CZE analysis was then performed to characterize the VLDL particles. Figure 2 shows the electropherograms of oxidized VLDL particles. For each donor, the mobility of oxidized VLDL peak was higher than that of native VLDL. The results indicated that it had a higher negative charge [23,24]. For each donor, the average peak area ratios (A_234_/A_214_ and A_280_/A_214_) of ox-VLDL were higher than those of native VLDL (Tables 1 and 2). The results suggested that it had higher levels of conjugated dienes and a higher protein to lipid ratio due to oxidation. But, some oxidized lipids such as dihydroxy-eicosatetraenoic acid, which has three conjugated double bonds with absorbance at 280 nm might also show higher A_280_/A_214_ ratio.

Absorbance of 234 nm was measured because some conjugated dienes formed on the polyunsaturated fatty acids of phospholipids in the process of oxidation [25]. The mechanisms of *in vivo* lipoprotein oxidation are not established yet. However, it is generally accepted that the oxidation involves a free radical process. First, the polyunsaturated fatty acid loses a hydrogen radical and molecular rearrangement occurs to form a conjugated diene. Then, the molecular radical formed takes up oxygen to form a peroxy radical, abstracts a hydrogen radical from an adjacent fatty acid and forms a hydroperoxide. Lipid peroxides can neutralize the positive charged side chain on lysine residues of apo B-100 and therefore increase the negative charge of LDL [23,24].

Diabetic patients often have high levels of blood glucose. To monitor *in vivo* VLDL particles under hyperglycemic conditions, native VLDL particles were *in vitro* modified by glucose (60 mM). CZE analysis was then performed to analyze the modified VLDL particles. Figure 3 shows the electropherograms of glycated VLDL particles. For each donor, the mobility of the glycated VLDL peak was similar to that of the native VLDL. For each donor, the average peak area ratios (A_234_/A_214_ and A_280_/A_214_) of glycated VLDL were also similar to those of native VLDL (Tables 1 and 3). This suggests that both particles had similar levels of conjugated dienes and protein to lipid ratios.

Although other measurements for glycosylation of VLDL have not been performed, we have previously analyzed *in vitro* glycated LDL and HDL particles [26,27]. For LDL particles, it was observed that glycated LDL had similar effective mobilities to native LDL. But, the peak area ratios (A_234_/A_214_, A_280_/A_214_) were slightly higher than those of native LDL, indicating low levels of oxidation and degradation of glycated LDL particles. For HDL particles, it was observed that glycated HDL had much lower effective mobilities than native HDL. In the reaction of glycosylation, Schiff base products formed between carbonyl groups of glucose and amine groups of apolipoproteins on HDL, which increased the molecular weight without changing the charges of HDL. Thus, the mobilities of HDL particles became lower, but the peak area ratios (A_234_/A_214_, A_280_/A_214_) were similar to native HDL. Kennedy *et al.* suggested that *in vivo* glycosylation led to the alteration of catabolisms of HDL and LDL, but the catabolisms of HDL appeared to be more accelerated than LDL [28]. Since LDL particles were hydrolyzed from VLDL particles *in vivo*, the effects of glycosylation on both particles were probably similar.

### 2.2. SDS-PAGE Analysis and Measurement of Cholesterol, Triglyceride and Protein Concentrations of VLDL Fractions

**Figure 4:**
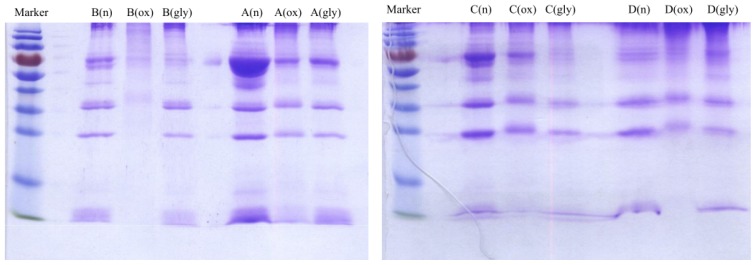
Sodium dodecyl sulfate polyacrylamide gel electrophoresis (SDS–PAGE) analysis of native (n), *in vitro* oxidized (ox) and glycated (gly) VLDL fractions of four healthy donors.

Concentrations of cholesterol, triglyceride and protein were measured for VLDL fractions as shown in Table 4. Each VLDL fraction was also analyzed by sodium dodecyl sulfate polyacrylamide gel electrophoresis (SDS-PAGE) as shown Figure 4. The protein concentrations were correlated with the SDS-PAGE analysis. For example, for Donor B, the measured protein concentrations were 2213, 872 and 1932 μg/mL for native, oxidized and glycated VLDL, respectively. On the SDS-PAGE gel, the band intensity was the highest for native VLDL and the lowest for oxidized VLDL. The degradation/fragmentation pattern of protein by SDS-PAGE analysis was also correlated with the peak area ratio (CPA ratio, A_280_/A_214_) in Tables 1–3. Since absorbance at 280 nm represented proteins, and absorbance at 214 nm represented all lipids, which had carbon-carbon double bonds, the peak area ratio (A_280_/A_214_) represented protein to lipid ratio. During the process of oxidation, lipoprotein particles became smaller due to lipid decomposition, and thus the protein to lipid ratios became larger as shown for each donor in Table 2.

### **2.**3. MEKC Profiles of Native, *in Vitro* Oxidized and Glycated VLDL Phospholipids

In human plasma, phospholipids are crucial components of lipoproteins. Oxidized phospholipids that are generated from the oxidation of lipoproteins are pro-inflammatory. These oxidized phospholipids are produced by the stimulation of potent oxidants. Some of them have been shown to be biological active, binding to endothelial cells and causing cell dysfunction, thus it is important to investigate phospholipids [29–31]. However, the peak mobilities and areas of MEKC profiles of phospholipids should not be compared with the corresponding VLDL particles, since phospholipids are only a small part of a VLDL particle, and mobility is determined by its charge to size ratio.

**Figure 5:**
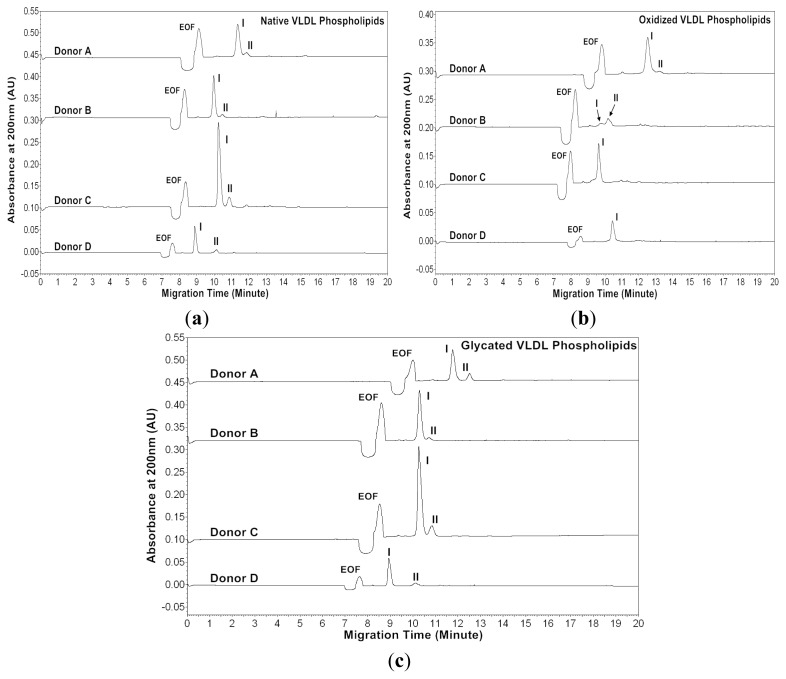
MEKC profiles of (**a**) native, (**b**) *in vitro* oxidized and (**c**) *in vitro* glycated VLDL phospholipids of four healthy subjects measured at absorbance 200 nm. CE voltage and temperature used were 25 kV and 40 °C, respectively. A pressure of 0.5 psi and 32 s injection were applied. The separation buffer consisted of 50 mM bile salts + 10 mM PB + 30% 1-propanol, pH 8.5. Sample buffer: 100 mM NaCl + 20% 1-propanol.

**Figure 6:**
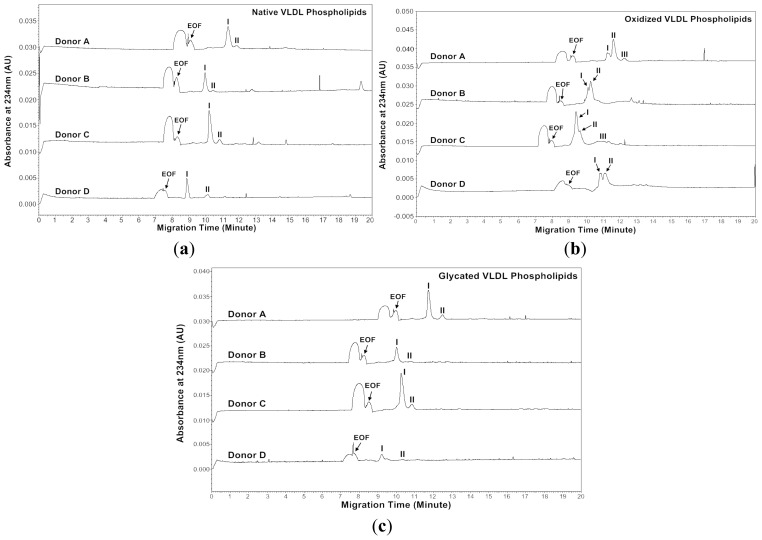
MEKC profiles of (**a**) native, (**b**) *in vitro* oxidized and (**c**) *in vitro* glycated VLDL phospholipids of four healthy subjects measured at absorbance 234 nm. MEKC conditions are the same as Figure 4.

Recently, we have developed a MEKC with a high-salt stacking method [32]. In this study, we analyzed phospholipids isolated from native, *in vitro* oxidized and glycated VLDL particles using the MEKC with high salt-stacking method. Two chloroform and two methanol fractions were collected from the solid-phase extraction (SPE) procedure. Since it was found that phospholipids were mostly in the first methanol fraction from our previous study, this fraction was used for the MEKC analysis [27]. Figures 5 and 6 show the MEKC profiles of native, oxidized and glycated VLDL phospholipids at absorbances 200 and 234 nm, respectively. Tables 5–10 show the effective mobilities and areas of the VLDL phospholipid peaks at absorbances 200 and 234 nm, respectively.

At absorbance 200 nm, the native VLDL phospholipids showed a major peak and a minor peak for each donor (Figure 5a). The oxidized VLDL phospholipids showed different profiles compared to those of the native VLDL phospholipids except for Donor A (Figure 5b). For Donor A, the oxidized VLDL phospholipids showed a major and a minor peak with similar mobilities to the native VLDL phospholipids. For Donor B, two smaller peaks appeared after oxidation, possibly due to lipid decomposition. It was suggested that when copper ions oxidized lipoproteins, they first reacted with pre-existing lipid hydroperoxides to produce the initiating radicals. The next events were loss of antioxidants, lipid peroxidation and decomposition of lipid hydroperoxides to produce reactive aldehydes [33,34]. For Donors C and D, one major peak with mobility similar to the major peak of native VLDL phospholipids remained, but the area reduced, possibly also due to lipid decomposition (Tables 5 and 6).

The glycated VLDL phospholipids also showed a major and a minor peak similar to the native VLDL phospholipids (Figure 5c). However, the mobilities of Donors A and C were more positive than the native VLDL phospholipids possibly due to phospholipid-linked advanced glycation end products (AGEs). The cross-linking increased the molecular weight without changing the charges of phospholipids, and thus resulted in lower mobilities (Tables 5 and 7) [35–37].

Absorbance of 234 nm was measured because some conjugated dienes formed on the polyunsaturated fatty acids of phospholipids in the process of oxidation as described in Section 2.1 [23,24]. At absorbance 234 nm, the native VLDL phospholipids showed a major peak and a minor peak for each donor (Figure 6a). Surprisingly, the major peak of oxidized VLDL phospholipids was resolved as two peaks for each donor (Figure 6b). The glycated VLDL phospholipids also showed a major peak and a minor peak similar to the native VLDL phospholipids (Figure 6c). Similar to absorbance at 200 nm, the mobilities of Donors A and C were more positive than the native VLDL phospholipids, possibly because of advanced glycation end products (AGEs) formed on phospholipids (Tables 8 and 10) [35–37].

Hydroperoxy, hydroxy, oxo and conjugated dienes formed on the polyunsaturated fatty acids of phospholipids during oxidation [24]. Phospholipids with conjugated dienes had a stronger absorbance at 234 nm, and the phenomenon was seen in Figure 6b. Under non-stacking conditions, each peak could not be resolved as two peaks at absorbance 234 nm (data not shown). Thus, our MEKC with high-salt stacking analysis could easily distinguish between native and oxidized VLDL phospholipids.

In summary, to analyze VLDLs from patients in the future, it should be helpful to use both CZE for VLDL particles and MEKC for VLDL phospholipids. The combination might also be useful for the further study concerning the role of oxidized and glycated VLDLs in the development of metabolic syndrome and diabetes.

### 2.4. Measurement of Thiobarbituric Acid Reactive Substances (TBARS assay) for VLDL fractions

Thiobarbituric acid reactive substances (TBARS) assay was carried out to monitor lipid peroxidation for native, oxidized and glycated VLDL fractions for each donor. Malondialdehyde (MDA) was a product of lipid peroxidation. The MDA-TBA adduct formed by the reaction of MDA and TBA was measured at absorbance 530 nm. The results of the TBARS assay is shown in Table 11. For each donor, oxidized VLDL had the highest levels of MDA, indicating that it contained the highest amount of lipid peroxides. Glycated VLDL had the lowest levels of MDA. The observation that native VLDL had slightly higher levels of MDA than glycated VLDL was probably due to autooxidation of native VLDL during the process of storage.

The peak area ratio (CPA ratio, A_234_/A_214_) in Tables 1–3 indicated the conjugated diene levels for native, oxidized and glycated VLDL. Conjugated dienes formed as a result of the oxidation of polyunsaturated lipids. The CPA ratio (A_234_/A_214_) was the highest for oxidized VLDL particles. The CPA ratios (A_234_/A_214_) for native and glycated VLDL particles were similar. The peak area (A_234_) in Tables 8–10 indicated conjugated diene levels in VLDL phospholipids. The oxidized VLDL phospholipids also contained the highest levels of conjugated dienes. Although MDA and conjugated dienes were different lipid oxidation products, both showed the highest concentrations in oxidized VLDL for each donor.

## 3. Experimental Section

### 3.1. Chemicals

The chemicals used in this study were: bile salts (50% sodium cholate and 50% sodium deoxycholate; Sigma Chemical, St. Louis, MO, USA), chloroform (CHCl_3_; Mallinckrodt Baker, Phillipsburg, NJ, USA), copper(II) sulfate pentahydrate (CuSO_4_·5H_2_O; Riedel-de Haën, Germany), deionized water (Millipore Simplicity; Millipore, Billerica, MA, USA), d-(+)-glucose (C_6_H_12_O_6_; Sigma Chemical), ethylenediaminetetraacetic acid (EDTA; Sigma Chemical), methanol (CH_3_OH; Echo Chemical), phosphate buffered saline (PBS; Sigma Chemical), phosphoric acid 85% (H_3_PO_4;_ Riedel-de Haën), potassium bromide crystal (KBr; J.T. Baker, Phillipsburg, NJ, USA), 1-propanol (C_3_H_7_OH; Sigma Chemical), sodium chloride (NaCl; Sigma Chemical), sodium hydroxide (NaOH; Riedel-de Haën), sodium phosphate dibasic (Na_2_HPO_4_; Sigma Chemical), and sodium phosphate monobasic (NaH_2_PO_4_; Sigma Chemical).

### 3.2. Healthy Subjects

The four healthy human blood plasma samples were kindly provided by Taichung Blood Donation Center (Taichung, Taiwan). Informed consent was obtained from each participant. This study was approved by National Changhua University of Education and Taichung Blood Center (Taichung, Taiwan).

### 3.3. Separation of Human VLDL Fractions by Ultracentrifugation

Isolation of lipoprotein fractions was performed using a Beckman Coulter Optima™ XL-100K following our previous procedure [26]. VLDL (*d* = 0.95–1.006 g/mL) was isolated from the plasma of four healthy donors by sequential ultracentrifugation. The collected VLDL fractions were immediately used for reaction and separation, otherwise kept at 4 °C for 2–3 weeks.

### 3.4. Preparation of Native VLDL Samples for CE Analysis

After the ultracentrifugation procedure, the KBr solution of a 800 μL VLDL fraction was exchanged with a 5 mM sodium phosphate solution using a 100 kDa ultrafiltration (UF) filter (Amicon, Micron Centrifugal Filter Devices, Ultracel YM-100; MW cut-off: 100,000, Millipore, Bedford, MA, USA). Finally, the VLDL solution was reconstituted to 200 μL in 5 mM sodium phosphate buffer, pH 7.40 for CE analysis.

### 3.5. *In Vitro* Oxidation of VLDL by Cu^2+^

After the ultracentrifugation procedure, the KBr solution of 800 μL VLDL fraction was exchanged with PBS buffer using a 100 kDa UF filter. The final volume of VLDL was reconstituted to 400 μL. Subsequently, a 400 μL of 5 μM CuSO_4_ solution was mixed with the solution, so the final concentration of Cu^2+^ was 2.5 μM. The VLDL solution mixture was then incubated in a 37 °C water bath and shaken at 80 rpm for 18 h. To quench the oxidation reaction, 0.0009 g of EDTA was added to the solution. In order to perform CE analysis, the buffer of the VLDL sample was exchanged with the oxidation solution mixture to 5 mM sodium phosphate buffer using a 100 kDa UF filter. The final volume of VLDL was reconstituted to 200 μL. CE analysis was then carried out.

### 3.6. *In Vitro* Glycation of VLDL by Glucose

A 400 μL VLDL solution in PBS buffer was prepared as in the previous section. Then, 400 μL of 120 mM glucose solution (in PBS buffer) and 0.0009 g EDTA were mixed with the VLDL solution, so the final glucose concentration was 60 mM. The solution was then incubated in a 37 °C oven for 5 days. Finally, the buffer of VLDL was exchanged to 5 mM sodium phosphate using a 100 kDa UF filter. The volume of VLDL was reconstituted to 200 μL. CE analysis was then carried out.

### 3.7. Liquid-Liquid and SPE of VLDL Phospholipids

The two lipid extraction procedures followed our previous study [27]. First, the final volume of the above-described native, oxidized or glycated VLDL sample was reconstituted to 800 μL. For liquid-liquid extraction, 0.75 mL methanol/chloroform (2:3 *v*/*v*) was mixed with the VLDL sample. The VLDL solution was vortexed for 3 min, and then centrifuged at 5000 rpm for 10 min. Subsequently, the lower organic phase was separated from the upper aqueous layer. The procedure was repeated another time using 1 mL methanol/water (1:1 *v*/*v*). The two lower organic phases were combined and dried under a nitrogen gas. A 1 mL of chloroform was then added to dissolve the lipids. Subsequently, SPE was carried out to separate phospholipids from neutral lipids. A Sep Pak cartridge column (Sep-Pak light silica cartridge, particle size: 55–105 μm, pore size: 125 Å, silica mass: 120 mg, column hold-up volume, 0.4 mL, Waters, Milford, MA, USA.) was used for SPE. Briefly, (a) the column was rinsed with 2 mL chloroform, (b) the 1 mL sample was slowly loaded onto the column, (c) 4 mL chloroform was added onto the column to elute neutral lipids, and two fractions were collected each with 2 mL, (d) 4 mL methanol was subsequently added onto the column to elute polar lipids. Again, two fractions were collected each with 2 mL. The first methanol fraction was then used for MEKC analysis.

### 3.8. CZE Analysis of VLDL Particles and MEKC Analysis of VLDL Phospholipids

The CE analysis was performed using a Beckman P/ACE MDQ capillary electrophoresis system (Beckman Instruments, Fullerton, CA, USA). The instrument has a diode-array detector and capillary cartridge coolant tubing. An IBM Pentium 4 computer was connected with the CE instrument. A 32 Karat software (version 8.0; Beckman Instruments: Fullerton, CA, USA) was applied to analyze electropherograms. In this study, uncoated fused-silica capillaries (i.d. 76 μm; o.d. 364 νm, Polymicro Technologies, Phoenix, AZ, USA) were used. The total and effective lengths of the capillary were 60.2 cm and 50.0 cm, respectively. The window width of the capillary was 2.0 mm. To activate a new capillary, it was sequentially rinsed with 1 N NaOH for 2 min, 0.1 M NaOH for 10 min, and deionized water for 10 min. Every day before analysis, the activated capillary was also rinsed with 1 N NaOH for 1 min, 0.1 N NaOH for 5 min, deionized water for 5 min, and CE separation buffer for 5 min. CE analysis was run from anode to cathode (normal polarity). Between runs, the capillary was routinely conditioned with 0.1 M NaOH for 2 min and deionized water for 2 min.

For VLDL particle analysis, CZE was performed. The CE voltage applied was 16 kV, and the capillary temperature was maintained at 25 °C. For sample introduction, the injection sequence was: (1) a 4-s pressure injection of deionized water with 0.4% (*v*/*v*) formaldehyde as the EOF marker, (2) a 4-s pressure injection of the VLDL sample and (3) a 4-s pressure injection of the CE separation buffer. A pressure of 0.5 psi was applied for sample injection. Both the sample buffer and CE separation buffer were 5 mM sodium phosphate, pH 7.40.

For VLDL phospholipid analysis, MEKC was carried out. The CE applied voltage was 25 kV, and the capillary temperature was kept at 40 °C. A 32-s pressure injection of sample was followed by a 4-s pressure injection of separation buffer. A pressure of 0.5 psi was applied for sample introduction. The sample buffer was 100 mM NaCl + 20% 1-propanol. The separation buffer was 50 mM bile salts + 10 mM PB + 30% 1-propanol, pH 8.5.

### 3.9. Analysis of Cholesterol, Triglyceride, Protein, MDA and SDS-PAGE

Concentrations of cholesterol and triglyceride of native VLDL fractions were measured by Changhua Christian Hospital (Changhua, Taiwan). Concentrations of proteins of native, oxidized and glycated VLDL fractions were measured by the Lowry’s method. Concentrations of MDA of native, oxidized and glycated VLDL fractions were measured by the TBARS assay kit (Abnova, Walnut, CA, USA). SDS-PAGE analysis was performed according to the standard procedure.

## 4. Conclusions

A simple and highly reproducible CZE method has been applied to analyze native, *in vitro* oxidized and glycated VLDL particles for four healthy subjects. The CE profiles of the three VLDL particles showed a major peak with good precisions of effective mobility and peak area. The mobility of the oxidized VLDL particle was higher than that of the native VLDL particle, indicating that it had a higher negative charge upon oxidation. Meanwhile, a newly developed MEKC method with high-salt stacking has been applied for the analysis of human VLDL phospholipids. The effective mobility and peak area showed good precisions. Native and *in vitro* oxidized VLDL phospholipids showed significantly different profiles at absorbance 234 nm. Furthermore, this method might be used to determine *in vivo* VLDL phospholipids from patients of MS and T2D.

## Figures and Tables

**Table 1:** The average effective mobility, peak area and peak area ratios of native VLDL particles analyzed by CZE (data are means ± SD of four replicates).

Native VLDL						

**Donor**	μ_eff_ (×10^−5^ cm2V^−1^S^−1^)	CV (%)	Area(214) (×10^4^)	CV (%)	CPA ratio (A_234_/A_214_) (×10^−2^)	CPA ratio (A_280_/A_214_) (×10^−2^)
A	−19.25 ± 0.14	0.75	129.39 ± 1.50	1.16	41.26 ± 0.46	17.98 ± 0.26
B	−16.59 ± 0.41	2.49	48.22 ± 0.50	1.04	38.68 ± 0.60	16.81 ± 0.17
C	−17.82 ± 0.23	1.29	109.48 ± 8.93	8.15	35.08 ± 4.08	17.59 ± 0.57
D	−20.51 ± 0.15	0.75	90.89 ± 0.77	0.85	43.52 ± 1.26	16.08 ± 0.27

**Table 2:** The average effective mobility, peak area and peak area ratios of *in vitro* oxidized VLDL particles analyzed by CZE (data are means ± SD of four replicates).

Oxidized VLDL						

**Donor**	μ_eff_ (×10^−5^ cm^2^V^−1^S^−1^)	CV (%)	Area(214) (×10^4^)	CV (%)	CPA ratio (A_234_/A_214_) (×10^−2^)	CPA ratio (A_280_/A_214_) (×10^−2^)
A	−25.68 ± 0.25	0.97	158.27 ± 2.44	1.54	63.76 ± 0.26	20.29 ± 0.60
B	−32.06 ± 0.07	0.23	72.72 ± 0.70	0.96	66.01 ± 0.92	22.26 ± 0.39
C	−31.61 ± 0.16	0.51	189.61 ± 10.65	5.62	72.74 ± 3.79	28.27 ± 2.68
D	−27.77 ± 0.12	0.42	169.34 ± 5.64	3.33	76.89 ± 0.47	23.81 ± 0.25

**Table 3:** The average effective mobility, peak area and peak area ratios of *in vitro* glycated VLDL particles analyzed by CZE (data are means ± SD of four replicates).

Glycated VLDL						

**Donor**	μ_eff_ (×10^−5^ cm^2^V^−1^S^−1^)	CV (%)	Area(214) (×10^4^)	CV (%)	CPA ratio (A_234_/A_214_) (×10^−2^)	CPA ratio (A_280_/A_214_) (×10^−2^)
A	−18.62 ± 0.36	1.91	129.09 ± 1.49	1.15	44.58 ± 0.13	16.78 ± 0.69
B	−17.69 ± 0.03	0.14	51.10 ± 2.66	5.20	38.14 ± 3.20	17.81 ± 0.67
C	−16.98 ± 0.10	0.57	105.25 ± 6.53	6.21	38.43 ± 3.56	18.42 ± 1.04
D	−20.47 ± 0.25	1.23	111.51 ± 4.35	3.90	46.73 ± 0.83	17.52 ± 1.45

**Table 4:** The concentrations of cholesterol, triglyceride and protein of native, *in vitro* oxidized and glycated VLDL particles (data are means of three replicates).

Donor	VLDL fractions	(mg/dL)	(mg/dL)	(μg/mL)

		Cholesterol	Triglyceride	Protein
A	native	62	236	6776
	oxidized			2605
	glycated			2128
B	native	45	78	2213
	oxidized			872
	glycated			1932
C	native	94	209	5221
	oxidized			2679
	glycated			867
D	native	231	272	10211
	oxidized			6717
	glycated			3835

**Table 5:** The average effective mobility and peak area of native VLDL phospholipids analyzed by MEKC at absorbance 200 nm (data are means ± SD of four replicates).

N-PLs				

	μ_eff_ (×10^−5^ cm^2^V^−1^S^−1^)	CV (%)	Area(200) (×10^4^)	CV (%)
Donor A				

Peak I	−4.49 ± 0.04	0.95	112.61 ± 4.27	3.80
Peak II	−5.30 ± 0.05	0.99	15.06 ± 1.32	8.78

Donor B				

Peak I	−4.25 ± 0.05	1.27	79.42 ± 6.87	8.65
Peak II	−5.23 ± 0.03	0.60	5.98 ± 0.20	3.35

Donor C				

Peak I	−4.63 ± 0.03	0.57	219.06 ± 6.11	2.79
Peak II	−5.70 ± 0.09	1.59	31.18 ± 0.64	2.04

Donor D				

Peak I	−3.97 ± 0.00	0.09	56.83 ± 1.84	3.23
Peak II	−6.85 ± 0.09	1.41	9.34 ± 0.25	2.69

**Table 6:** The average effective mobility and peak area of *in vitro* oxidized VLDL phospholipids analyzed by MEKC at absorbance 200 nm (data are means ± SD of four replicates).

Ox-PLs				

	μ_eff_ (×10^−5^ cm^2^V^−1^S^−1^)	CV (%)	Area(200) (×10^4^)	CV (%)
Donor A				

Peak I	−4.44 ± 0.02	0.42	116.27 ± 4.72	4.06
Peak II	−5.48 ± 0.29	5.27	14.16 ± 0.59	4.15

Donor B				

Peak I	−3.81 ± 0.03	0.84	10.66 ± 0.17	1.58
Peak II	−4.71 ± 0.06	1.26	26.75 ± 1.10	4.12

Donor C				

Peak I	−4.53 ± 0.02	0.52	84.41 ± 2.92	3.46

Donor D				

Peak I	−4.34 ± 0.03	0.68	49.96 ± 2.55	5.11

**Table 7:** The average effective mobility and peak area of *in vitro* glycated VLDL phospholipids analyzed by MEKC at absorbance 200 nm (data are means ± SD of four replicates).

Gly-PLs				

	μ_eff_ (×10^−5^ cm^2^V^−1^S^−1^)	CV (%)	Area(200) (×10^4^)	CV (%)
Donor A				

Peak I	−3.04 ± 0.02	0.60	120.52 ± 4.33	3.59
Peak II	−4.08 ± 0.05	1.17	17.30 ± 0.23	1.31

Donor B				

Peak I	−4.42 ± 0.02	0.49	80.01 ± 1.29	1.61
Peak II	−5.41 ± 0.02	0.31	5.40 ± 0.10	1.84

Donor C				

Peak I	−4.16 ± 0.02	0.53	227.95 ± 6.88	3.02
Peak II	−5.04 ± 0.03	0.58	33.81 ± 0.57	1.69

Donor D				

Peak I	−4.24 ± 0.04	0.95	56.99 ± 1.59	2.79
Peak II	−6.55 ± 0.15	2.23	8.31 ± 0.39	4.70

**Table 8:** The average effective mobility and peak area of native VLDL phospholipids analyzed by MEKC at absorbance 234 nm (data are means ± SD of four replicates).

N-PLs				

	μ_eff_ (×10^−5^ cm^2^V^−1^S^−1^)	CV (%)	Area(234) (×10^4^)	CV (%)
Donor A				

Peak I	−4.52 ± 0.05	1.02	6.96 ± 0.12	1.77
Peak II	−5.39 ± 0.05	0.90	1.06 ± 0.05	4.34

Donor B				

Peak I	−4.28 ± 0.01	0.30	4.11 ± 0.41	9.92
Peak II	not measurable			

Donor C				

Peak I	−4.69 ± 0.05	1.05	9.01 ± 0.25	2.80
Peak II	−5.79 ± 0.06	1.03	1.15 ± 0.01	0.89

Donor D				

Peak I	−4.09 ± 0.03	0.76	3.37 ± 0.13	3.78
Peak II	not measurable			

**Table 9:** The average effective mobility and peak area of *in vitro* oxidized VLDL phospholipids analyzed by MEKC at absorbance 234 nm (data are means ± SD of four replicates).

Ox-PLs				

	μ_eff_ (×10^−5^ cm^2^V^−1^S^−1^)	CV (%)	Area(234) (×10^4^)	CV (%)
Donor A				

Peak I	−3.94 ± 0.03	0.67	3.22 ± 0.17	5.20
Peak II	−4.46 ± 0.01	031	7.50 ± 0.28	3.80
Peak III	−5.36 ± 0.04	0.69	1.01 ± 0.05	5.47

Donor B				

Peak I	−3.93 ± 0.03	0.70	7.94 ± 0.04	0.47
Peak II	−4.23 ± 0.03	0.67	3.85 ± 0.05	1.39

Donor C				

Peak I	−4.03 ± 0.03	0.80	12.66±0.47	3.70
Peak II	−4.61 ± 0.03	0.67	5.00±0.12	2.38
Peak III	−5.85 ± 0.06–−8.39±0.05	0.97 0.54	8.87 ± 0.13	1.43

Donor D				

Peak I	−4.13 ± 0.01	0.33	4.35 ± 0.12	2.65
Peak II	−4.63 ± 0.02	0.52	8.50 ± 0.27	3.20

**Table 10:** The average effective mobility and peak area of *in vitro* glycated VLDL phospholipids analyzed by MEKC at absorbance 234 nm (data are means ± SD of four replicates).

Gly-PLs				

	μ_eff_ (×10^−5^ cm^2^V^−1^S^−1^)	CV (%)	Area(234) (×10^4^)	CV (%)
Donor A				

Peak I	−3.08 ± 0.02	0.52	7.42 ± 0.18	2.49
Peak II	−4.13 ± 0.02	0.59	1.20 ± 0.04	3.14

Donor B				

Peak I	−4.48 ± 0.04	0.99	3.52 ± 0.04	1.16
Peak II	not measurable			

Donor C				

Peak I	−4.19 ± 0.02	0.44	10.41 ± 0.25	2.43
Peak II	−5.06 ± 0.02	0.32	1.57 ± 0.04	2.68

Donor D				

Peak I	−4.31 ± 0.02	0.37	2.19 ± 0.19	8.68
Peak II	not measurable			

**Table 11:** The concentrations of MDA of native, *in vitro* oxidized and *in vitro* glycated VLDL fractions (data are one representive measurement).

Donor	Native (μM)	Oxidized (μM)	(Glycated) (μM)
A	0.56	1.16	0.39
B	1.86	2.06	0.92
C	1.46	2.94	0.58
D	3.17	10.58	1.09

## References

[1] Dominiczk M.H., Rifai N., Warnick G.R., Dominiczk M.H. (1997). Handbook of Lipoprotein Testing.

[2] Unger R.H., Clark G.O., Scherer P.E., Orci L. (2010). Lipid homeostasis, lipotoxicity and the metabolic syndrome. Biochim. Biophys. Acta.

[3] Brunzell J.D., Davidson M., Furberg C.D., Goldberg R.B., Howard B.V., Stein J.H., Witztum J.L. (2008). Lipoprotein management in patients with cardiometabolic risk. Diabetes Care.

[4] Moller D.E., Kaufman K.D. (2005). Metabolic syndrome: A clinical and molecular perspective. Annu. Rev. Med.

[5] Ginsberg H.N. (2000). Insulin resistance and cardiovascular disease. J. Clin. Invest.

[6] Adiel M., Olofsson S.O., Taskinen M.R., Boren J. (2008). Overproduction of very low-density lipoproteins is the hallmark of the dyslipidemia in the metabolic syndrome. Arterioscler. Thromb. Vasc. Biol.

[7] Dergunov A.D., Ponthieux A., Mel’kin M.V., Lambert D., Sokolova O.Y., Akhmedzhanov N.M., Visvikis-Siest S., Siest G. (2009). Capillary isotachophoresis study of lipoprotein network sensitive to apolipoprotein E phenotype. 1. ApoE distribution between lipoproteins. Mol. Cell Biochem.

[8] Zhang B., Bottcher A., maizumi S., Noda K., Schmitz G., Saku K. (2007). Relation between charge-based apolipoprotein B-containing lipoprotein subfractions and remnant-like particle cholesterol levels. Atherosclerosis.

[9] Dergunov A.D., Hoy A., Smirnova E.A., Visvikis S., Siest G. (2003). Charge-based heterogeneity of human plasma lipoproteins at hypertriglyceridemia: capillary isotachophoresis study. Int. J. Biochem. Cell Biol.

[10] Inano K., Tezuka S., Miida T., Okada M. (2000). Capillary isotachophoretic analysis of serum lipoproteins using a carrier ampholyte as spacer ion. Ann. Clin. Boichem.

[11] Bottcher A., Schlosser J., Kronenberg F., Dieplinger H., Knipping G., Lackner K.J., Schmitz G. (2000). Preparative free-solution isotachophoresis for separation of human plasma lipoproteins: Apolipoprotein and lipid composition of HDL subfractions. J. Lipid Res.

[12] Schlenck A., Herbeth B., Siest G., Visvikis S. (1999). Characterization and quantification of serum lipoprotein subfractions by capillary isotachophoresis: Relationships with lipid, apolipoprotein, and lipoprotein levels. J. Lipid Res.

[13] Zorn U., Wolf C.F., Wennauer R., Bachem M.G., Grünert A. (1999). Separation of lipoproteins by capillary isotachophoresis combined with enzymatic derivatization of cholesterol and triglycerides. Electrophoresis.

[14] Schmitz G., Möllers C., Richter V. (1997). Analytical capillary isotachophoresis of human serum lipoproteins. Electrophoresis.

[15] Schmitz G., Möllers C. (1994). Analysis of lipoproteins with analytical capillary isotachophoresis. Electrophoresis.

[16] Schmitz G., Borgmann U., Assmann G. (1985). Analytical capillary isotachophoresis: A routine technique for the analysis of lipoproteins and lipoprotein subfractions in whole serum. J. Chromatogr.

[17] Macfarlane R.D., Bondarenko P.V., Cockrill S.L., Cruzado I.D., Koss W., McNeal C.J., Spiekerman A.M., Watkins L.K. (1997). Development of a lipoprotein profile using capillary electrophoresis and mass spectrometry. Electrophoresis.

[18] Ping G., Zhu B., Jabasini M., Xu F., Oka H., Sugihara H., Baba Y. (2005). Analysis of lipoproteins by microchip electrophoresis with high speed and high reproducibility. Anal. Chem.

[19] Wang H., Han C., Wang H., Cao L., Wang G. (2011). Simultaneous determination of high-density lipoprotein, very low-density lipoprotein and low-density lipoprotein subclass in human serum by microchip CE. Chromatographia.

[20] Agren J.J., Kurvinen J.P., Kuksis A. (2005). Isolation of very low density lipoprotein phospholipids enriched in ethanolamine phospholipids from rats injected with Triton WR 1339. Biochim. Biophys. Acta.

[21] Dashti M., Kulik W., Hoek F., Veerman E.C., Peppelenbosch M.P., Rezaee F. (2011). A phospholipidomic analysis of all defined human plasma lipoproteins. Sci. Rep.

[22] Hidaka H., Hanyu N., Sugano M., Kawasaki K., Yamauchi K., Katsuyama T. (2007). Analysis of human serum lipoprotein lipid composition using MALDI-TOF mass spectrometry. Ann. Clin. Lab. Sci.

[23] Sinclair A.J., Barnett A.H., Lunec J. (1990). Free radicals and antioxidant systems in health and disease. Br. J. Hosp. Med.

[24] Junko A., Migiwa A., Naoki Y., Hideyuki N., Yasuhiro U. (2006). Analysis of phosphatidylcholine oxidation products in human plasma using quadrupole time-of-flight mass spectrometry. Kobe. J. Med. Sci.

[25] Gieseg S.P., Estebauer H. (1994). Low density lipoprotein is saturable by pro-oxidant copper. FEBS Lett.

[26] Chiu C.H., Peng Y.N., Yang Y.L., Tsai M.H., Ho Y.L., Wu C.Y., Liu M.Y. (2008). *In vitro* oxidized and glycated human low-density lipoprotein particles characterized by capillary zone electrophoresis. J. Chrom. B.

[27] Wu C.Y., Peng Y.N., Chiu J.H., Ho Y.L., Chong C.P., Yang Y.L., Liu M.Y. (2009). Characterization of *in vitro* modified human high-density lipoprotein particles and phospholipids by capillary zone electrophoresis and LC ESI-MS. J. Chrom. B.

[28] Kennedy L., Baynes J.W. (1984). Non-enzymatic glycosylation and the chronic complications of diabetes: An overview. Diabetologia.

[29] Navab M., Ananthramaiah G.M., Reddy S.T., VanLenten B.J., Ansell B.J., Fonarow G.C., Vahabzadeh K., Hama S., Hough G., Kamranpour N. (2004). Thematic review series: The pathogenesis of atherosclerosis the oxidation hypothesis of atherogenesis: The role of oxidized phospholipids and HDL. J. Lipid. Res..

[30] Ewa N. (2005). Phospholipid mediators in the vessel wall: Involvement in atherosclerosis. Curr. Opin. Clin. Nutr. Metab. Care.

[31] Leitinger N. (2003). Oxidized phospholipids as modulators of inflammation in atherosclerosis. Curr. Opin. Lipidol.

[32] Chong C.P., Lin T.Y., Chang C.L., Yang Y.L., Tsai M.H., Yu Y.S., Liu M.Y. (2011). Micellar electrokinetic chromatography profiles of human high-density lipoprotein phospholipids. Electrophoresis.

[33] Steinbrecher U.P., Witztum J.L., Parthasarathy S., Esterbauer H. (1987). Decrease in reactive amino groups during oxidation or endothelial cell modification of LDL. Arteriosclerosis.

[34] Jialal I., Grundy S.M. (1992). Effect of dietary supplementation with alpha-tocopherol on the oxidative modification of low density lipoprotein. J. Lipid Res.

[35] Goldin A., Beckman J.A., Schmidt A.M., Creager M.A. (2006). Advanced glycation end products: Sparking the development of diabetic vascular injury. Circulation.

[36] Singh R., Barden A., Mori T., Beilin L (2001). Advanced glycation end products: A review. Diabetologia.

[37] Bucala R., Makita Z., Vega G., Grundy S., Koschinsky T., Cerami A., Vlassara H. (1994). Modification of low density lipoprotein by advanced glycation end products contributes to the dyslipidemia of diabetes and renal insufficiency. Proc. Natl. Acad. Sci. USA.

